# Current Concepts in the Treatment of Early Onset Scoliosis

**DOI:** 10.3390/jcm13154472

**Published:** 2024-07-31

**Authors:** Alexandra N. Johnson, Robert K. Lark

**Affiliations:** Department of Orthopaedics, Duke University Medical Center, Durham, NC 27701, USA; anj28@duke.edu

**Keywords:** early onset scoliosis, congenital scoliosis, magnetically controlled growing rods, vertebral body tethering, casting

## Abstract

Despite many surgical advances in the treatment of early onset scoliosis (EOS) over the past two decades, this condition remains a challenge to address. While otherwise healthy children can have EOS, many of these patients have complicated comorbidities making proper treatment algorithms extraordinarily difficult. Non-operative measures can be successful when initiated early, but are many times utilized as a delay tactic until growth-friendly operative procedures can be safely performed. This article will summarize the current concepts in the treatment of EOS with a focus on the surgical advances that have recently been made.

## 1. Background

Early onset scoliosis is defined by the Scoliosis Research Society as a coronal curve measured on an AP radiograph of at least 10 degrees prior to age 10 regardless of etiology. This complex condition is quite heterogeneous with multiple etiologies, associated comorbidities, and differing phenotypes. A short list of causes can be found in Table 1. The varied nature and severity of the condition can make treatment decisions difficult. Despite the diversity in presentation, in 2014, Vitale et al. provided a classification system that can be helpful in systematically organizing the condition, allowing providers to categorize patients throughout their clinical course prior to treatment intervention [1]. The final classification consisted of a continuous age prefix, etiology (congenital or structural, neuromuscular, syndromic, and idiopathic), major curve angle (1, 2, 3, or 4), and kyphosis (−, N, or +) variables and an optional progression modifier (P0, P1, or P2). This classification has been validated with excellent interobserver reliability and can be utilized to standardize clinical and research communications [2]. While we have relatively clear indications for bracing and surgery in the adolescent population, these indications are less clear in EOS. We hope this article will provide a framework from which to base these difficult decisions centered on our best available literature.

## 2. Non-Operative Treatment

Casting has been a mainstay of treatment of early onset scoliosis since it was first described nearly 150 years ago by Sayre using a plaster jacket technique. It has been extensively modified through the years to the contemporary applications of casts popularized by Mehta, first in 1976. In her original 1980 series, the cure rate was 100% in patients under the age of 20 months with considerable palliative delay of over two thirds of the older patients [3]. Multiple studies further supporting extension, derotation, and flexion (EDF) casting techniques have been published but none has been able to replicate the success of Mehta’s cohort. Utilizing Mehta’s article as a guide, the indications for casting for infantile idiopathic casting include rib phase 2 or rib phase 1 with a rib vertebral angle difference of more than 20 degrees. Per 2016 SOSORT guidelines, casting is recommended to treat infantile scoliosis for curve stabilization. In infantile idiopathic scoliosis, cure rates of 45–49% have been reported with greater than 5-year follow-up with high levels of palliation in non-cured groups [4,5,6]. Cast application has been used in non-idiopathic early onset scoliosis as well. However, it is less effective at preventing definitive surgery than in idiopathic curves. Hence there are no clear indications for initiating casting in this cohort. Casting can delay the timing of index surgery in congenital, neuromuscular, and syndromic curves, which can be a critical factor in treating these populations. Various authors have reported delay of surgery of 26.5–39 months in non-idiopathic curves [7,8,9,10]. Success rates remain highest in idiopathic curves. Age and percent of coronal curve correction in cast are the most significant predictors of treatment success [11].

### Casting Technique

Casting techniques have been published with multiple variations including over the shoulder casting versus thorax and torso only, plaster versus fiberglass materials, traditional and waterproof cast padding materials, and traditional EDF versus cantilever bending, all effective in treatment. Fedorak demonstrated no significant differences in casts placed over the shoulder compared to those without shoulder straps [12]. Shoulder straps may be useful in challenging cases with a more proximal apex, severe kyphosis, or other patient factors that may be improved by a more controlling cast.

Traditional EDF casting presents equipment challenges for some institutions where traditional Risser or Cotrel frames are not available. As such, some novel modifications have been made to the casting technique. One such modification is the use of an OSI Jackson table with padded dowel rods at the shoulder and sacrum levels with cranial and caudal traction applications [13]. Additional modifications are known including cantilever bending through a suspensory technique.

Finally, reports of neurotoxic effects of anesthetics in young children have prompted concern about the effect of repeat anesthetic events in this population. While there remains equipoise over the risk versus benefit relationship associated with repeated anesthetic events in this population, new experience has shown that awake casting may offer an effective and safe option. In a recent comparative study, patients who underwent awake casting had ≥10° of improvement in the major curve from baseline to casting cessation in 72.4% of awake casting participants. Awake patients presented similar radiographic outcomes with regard to major curve correction, as well as gain in thoracic and total spine height, when compared to those who were casted under general anesthesia [14].

## 3. Bracing

Bracing is often used as an adjunct to casting in the treatment of early onset scoliosis and follows similar principles as casting. Per SOSORT guidelines, bracing is recommended to attempt to delay or avoid surgery.

Favorable results have been reported by authors utilizing computer-aided design and manufacturing (CAD/CAM)-generated elongation bending derotation braces to apply the same three-dimensional correction of casting without the drawbacks of repeat anesthetic events. In this report of 12-month outcomes, the juvenile group had 23% correction, 47% stabilization, and 30% progression of curves. The infantile group had 50% correction, 32% stabilization, and 18% progression of curves. The same author published 3.4-year average follow-up from nine patients which showed encouraging results. Four patients were fully corrected with serial bracing alone (curve ≤ 10 degrees). Five patients with more rigid curves showed improvement from mean 57 degrees to mean 21 degrees (10 to 44 degrees) [15,16]. Similar intermediate-term results have been reported by other authors with progressive improvement of idiopathic infantile and stability of congenital and neuromuscular patients with bracing regimens [17]. Idiopathic patients demonstrated improvements 74% of the time with an average of five years of follow-up. We can anticipate a higher evidence level soon as there is a multicenter, prospective trial underway directly comparing casting and bracing in idiopathic early onset scoliosis [18].

While few studies directly address bracing as a primary and independent treatment modality, this early evidence suggests that bracing can be useful in patients who are poorly tolerating the constant wear burden of casts as they can be donned and doffed freely. This does, however, rely on patient compliance. Brace compliance and wear rates have not been studied in this population.

Brace styles vary and can include, but are not limited to, postural support braces such as the Spio vest or SureStep TLSO, symmetric braces such as the Boston brace, asymmetric derotational braces such as the Rigo-Cheneau, ScoliOlogiC^®^, or Gensingen-Cheneau, or nighttime bending braces such as the Providence and Charleston braces, as well as the previously described elongation bending derotation braces. In the adolescent idiopathic population, some superiority of the Rigo Cheneau brace has been identified with RCOs having lower mean and percent major curve progression versus those with TLSOs [19]. Limited definitive evidence is available to promote one type of brace over others in the early onset population and it should be implemented using surgeon judgement and shared decision making with parents.

While there are studies in the adolescent population evaluating scoliosis-specific exercise programs such as the Schroth method and techniques of the Postural Restoration Institute, to the authors’ knowledge, there is no known data for the implementation of physical therapy programs in the treatment of early onset scoliosis.

## 4. Preoperative Evaluation

Comprehensive awareness and optimization of comorbidities are essential in this unique population and should begin with a detailed personal medical and birth history, family history, and detailed physical exam. Pregnancy and birth history should be solicited with special attention to prenatal care, screening exam findings, and antenatal and postnatal events such as intensive care needs. Further history should be obtained including evaluation of growth curve and motor and neurologic developmental milestones. Any previous illness, hospitalizations, surgeries, and identified comorbid conditions of the CNS, renal–urologic, cardiac, and endocrine systems should be noted. Focused history should be obtained including the age of onset, known history of progression, and previous intervention. Physical examination should be thorough and include evaluation of general appearance and nutrition status, gait, extremity deformity, chest wall or rib deformities, neurologic function, and cutaneous signs of spinal dysraphism.

Initial imaging studies in EOS should begin with low-dose posterior–anterior and lateral X-rays. Biplanar slot scanning imaging should be used whenever possible to minimize cumulative radiation exposure. These initial radiographs may reveal congenital abnormalities including associated chest wall and rib deformities. Advanced imaging including whole spine MRI is typically recommended and should be carefully considered to evaluate for neuraxial pathology and abnormalities of the craniocervical junction. A recent multicenter, registry-based international study reports intrathecal abnormalities such as tethered spinal cord, syrinx, and Chiari malformation in 24% of 836 early onset scoliosis patients evaluated by MRI [20]. In addition to spine MRI, additional MRI studies have been described to quantify regional dynamic thoracic function through the use of quantitative dynamic thoracic MRI [21,22]. Computed tomography can be useful in preoperative planning for definition of bony architecture, especially in the congenital scoliosis population, and for implementation with various proprietary intraoperative navigation systems.

Regarding congenital scoliosis, failure of formation and segmentation during somitogenesis has been proposed as the primary mechanism. As such, screening of other congenital anomalies, especially of renal, cardiac, and lung, should be performed. Tracheo-esophageal fistula can present as failure to thrive. Anorectal anomalies are detected in the neonatal period only. Associated anomalies have been identified to occur in up to sixty percent of cases [23,24]. Renal anomalies such as horseshoe kidney, renal aplasia, and duplicate ureters can be seen in 20% of cases. Twenty-five percent of patients have associated cardiac defects including VSD, ASD, tetralogy of Fallot, and transposition of arteries. Cardiac and renal screening ultrasounds are indicated.

Previous studies have described restrictive and obstructive lung disease in children with early onset scoliosis. One spirometric measure, forced vital capacity (FVC), reflects restrictive changes and is a measure of respiratory reserve. It is expressed as a percentage of normal, using arm span or ulnar length to estimate height in the absence of spine curvature [25,26]. PFTs represent a challenge to children under the age of 5 and those with developmental delay precluding performance of the test. Surrogates have been investigated including the 6 min walk test, however, no reliable surrogate has been identified [27]. Other pulmonary evaluations can include total body plethysmograph or gas dilution techniques. Sleep studies can also be helpful as up to 92% of children with thoracic insufficiency related to congenital scoliosis have been found to have sleep apnea [28].

Prior to surgical intervention, consideration should be given to optimizing any comorbidities as well as obtaining a comprehensive estimate of the risks of scoliosis surgery in the context of the patient’s condition and existing medical comorbidities such as using the previously mentioned C-EOS. A multidisciplinary team approach to ensure appropriate screening studies are performed and any medical comorbidities optimized, especially pulmonary and nutrition, is beneficial.

## 5. Operative Treatment

Surgical treatment options in early onset scoliosis seek to apply an individualized approach and, upon failure of conservative therapies, optimize growth-friendly techniques. Surgical approaches are generally classified into three types according to the amount of correction force applied: distraction-based (both spine and rib based), compression-based growth modulation, and growth guidance systems [29]. In certain cases, primary definitive arthrodesis is appropriate.

Prior to proceeding with growth-preserving surgeries, some patients may benefit from a period of halo gravity traction (HGT). HGT has been shown to allow severe and stiff curves to achieve correction at a rate similar to small, more flexible curves without HGT with 22–40% correction observed [30,31]. Typical HGT protocols include traction weight at one pound per kilogram of body weight or roughly 40–50% body weight.

Indications for surgical intervention vary based upon etiology of the deformity and some variability still exists. In idiopathic EOS, surgical treatment is indicated in curves exceeding 60 degrees or with a failure of conservative treatment with rapid progression of deformity [32]. When considering timing of “growth-friendly” surgery, an assessment of the peak growth velocity, remaining spinal growth, and risk of curve progression is required. Delaying surgical treatment whenever possible is preferred as a complication reduction strategy as each year of delay of instigation of traditional growing rod surgery reduces the total complication rate by 13% [33]. In neuromuscular EOS, surgery is indicated for severe deformities responsible for costo-pelvic impingement, functional or pulmonary impairment, pain, or limitations to hygiene. Scoliosis surgery can be very rewarding and offer considerable improvements to quality of life, cardiopulmonary function, sitting balance and positioning, and physical appearance [34]. Medical optimization and multidisciplinary care are essential in the perioperative period as this population is vulnerable to complications. Further considerations should be given to syndromic patients, especially to those with connective tissue disorders or metabolic bone disease in anticipating conservative treatment failures and implant selection. Finally, surgery should be considered in congenital scoliosis upon rapid progression of the curve or the expectation of rapid progression secondary to the type and number of anomalies.

## 6. Distraction-Based Methods

Distraction-based systems correct spinal deformities by mechanically applying a distractive force across the deformed segment of the spine with anchors at the top and bottom of the implants, which commonly attach to the spine, rib, and/or the pelvis.

Traditional growing rods, TGRs, have evolved since their original inception by Harrington in 1962. These evolved from single rod constructs to dual rod constructs with improved results [35,36]. High complication rates have been cited due to the need for repeated lengthening. Complications include implant failure, wound complications, infection, and autofusion of the spine. Performing surgical lengthening at intervals of 6 months led to significantly greater growth and correction than those lengthened less frequently, however, each additional surgery increases the likelihood of complications by 24%. Traditional growing rods have decreased in utilization since the arrival of magnetically controlled growing rods (MCGRs). Limitations in the MCGR device do make it inappropriate for patients with kyphosis or short stature, thus, TGRs still have a valuable role in treatment [37].

## 7. Magnetically Controlled Growing Rods

**Figure 1:**
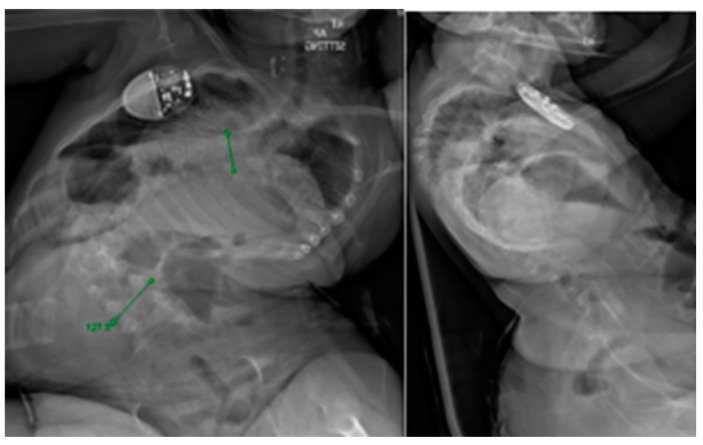
Preoperative sitting PA and lateral views of a 5-year-old female with cerebral palsy and associated neuromuscular scoliosis.

**Figure 2:**
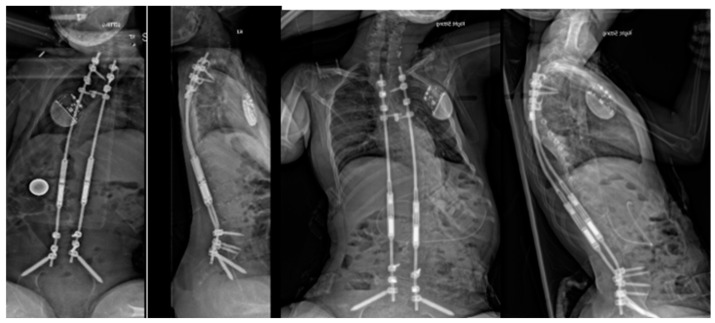
Postoperative radiographs of the same patient at 6 weeks and 3 years postimplantation of magnetically controlled growing rods with sustained curve control and excellent sitting balance.

Originally described by Takaso in 1998, MCGRs were developed to address the complications that accompanied TGR lengthenings [38]. After initial implantation, patients are lengthened at short-term intervals in the clinic setting using an external remote control (ERC), eliminating the need for open lengthenings. MCGRs were shown to have similar treatment outcomes with respect to deformity correction and thoracic height, however, the device does have a notable complication profile of its own and requires careful patient selection for optimal use [39]. Various complications have been reported with MCGRs including implant or ERC malfunction and implant breakage or loosening with up to 33% revision rates [40,41]. Recently, concerns over metallosis have been brought forth with histologic changes of phagocytic cells seen in patients undergoing rod exchange with no long-term implications understood at this point [42]. Concerns remain over neurotoxic implications of increased serum titanium as it does cross the blood–brain barrier. Recent studies have suggested rates of unplanned return to the operating room within two years of implantation as high as 40% [43]. For these reasons, the device was suspended in certain countries. Only recently in February 2024 has the system been re-instated in the United Kingdom. Despite its imperfect execution, the implant is widely used. Careful patient selection is necessary when considering MCGRs. Per FDA and manufacturer guidelines, MCGRs are to be implanted only in skeletally immature patients less than 10 years of age with severe progressive spinal deformities associated with or at risk of thoracic insufficiency syndrome (TIS). A clinical example is shown pre-operatively in Figure 1 and post-operatively in Figure 2. MCGRs are inappropriate for patients with insufficient spinal height, inadequate skin and soft tissue cover, a stiff spinal curve, a sagittal curve apex above T3, hyperkyphosis, or patients requiring repetitive MRI [44]. Caution should be exercised in patients with increased soft tissue envelopes and BMI over 25, for example, those with Prader–Willi, which could preclude the transmission of magnetic impulses. Per manufacturer instructions for use, each MCGR unit should be implanted for no longer than 2 years. Further safety information and instructions for proper use are available on the manufacturer’s website and should be carefully studied by implanting surgeons.

Finally, the “law of diminishing returns” was initially used to describe the phenomenon of the inability of the spine to continue lengthening over repeated lengthenings of traditional growing rods [45]. Recently, this issue has been described to plague MCGRs as well with only 21.7% of rods expanding to within 80% of the maximum excursion. In this study, patients with primary, secondary, and conversion MCGRs were evaluated by radiographic lengthening. Initial MCGRs were noted to deploy longer distances compared to revision implants. Further, they found improved excursion in the 90 mm actuators [46].

When considering TGRs and MCGRs regarding health-related quality of life issues, some evidence demonstrates a modest benefit to MCGRs with decreased economic burden and overall higher satisfaction with MCGRs and well as improvements in pain, emotion, child satisfaction, and parent satisfaction albeit without statistical significance [47]. Despite their limitations, MCGRs have become a preferred implant in the treatment of EOS with over 80% of index EOS cases implanting MCGRs [48].

## 8. VEPTR

Developed in 1989 by Dr. Robert Campbell, the vertical expandable prosthetic titanium rib (VEPTR) was designed to treat congenital scoliosis with thoracic insufficiency via expansion thoracostomy [49]. The device is a modular longitudinal rib-based distraction device, which is used as a rib-to-rib construct, a hybrid construct (rib-to-lumbar hook), or a rib-to-pelvis construct. This opening wedge thoracostomy is primarily intended to increase thoracic cavity volume with any secondary effects on the spinal deformity, however, the VEPTR has been described in use without opening wedge thoracoplasty for scoliosis without rib deformity. Early enthusiasm for the VEPTR has since tempered with the increasing use of magnetically controlled growing rods and reports of high complication rates, up to 163% in thoracic insufficiency patients, as well as high rates of complications in congenital scoliosis without rib deformity [48,49,50,51]. Further, early positive results on overall spinal growth and space available for lungs have not been reproduced although El –Hawary et al. have published a 79% rate of overall improvement of scoliosis as well increased total spine height in their review of 35 patients [50].

## 9. Spring Distraction

In an effort to address the drawbacks of distraction-based implants including the inability to mimic continuous physiological spinal growth [52], stiffness, and the “law of diminishing returns”, as well as implant failures and expense, the spring distraction system (SDS) was developed. The SDS consists of one or more compressed springs positioned around a standard sliding rod to provide active continuous distraction of the spine to stimulate growth and further correction. Early prospective data demonstrate an average 51% reduction of curve with this device as well as low adverse events or unplanned returns to the operating room at a rate of 0.1 per patient per year [53].

**Figure 3:**
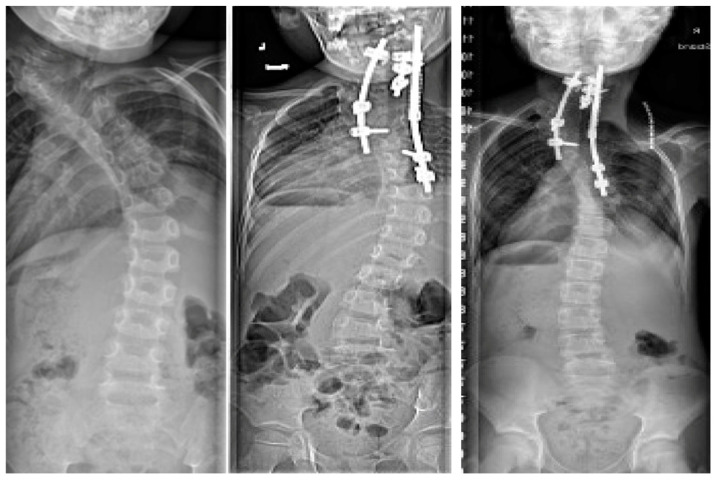
Preop, immediate postop, and 6 years postop with gradual correction with the spring distraction system.

Child with Goldenhar syndrome and severe cervicothoracic kyphoscoliosis at preop, immediate postop, and 6 years postop with gradual correction with the spring distraction system in Figure 3 (images courtesy of Dr. RM Castelein).

## 10. Growth Modulation Methods

**Figure 4:**
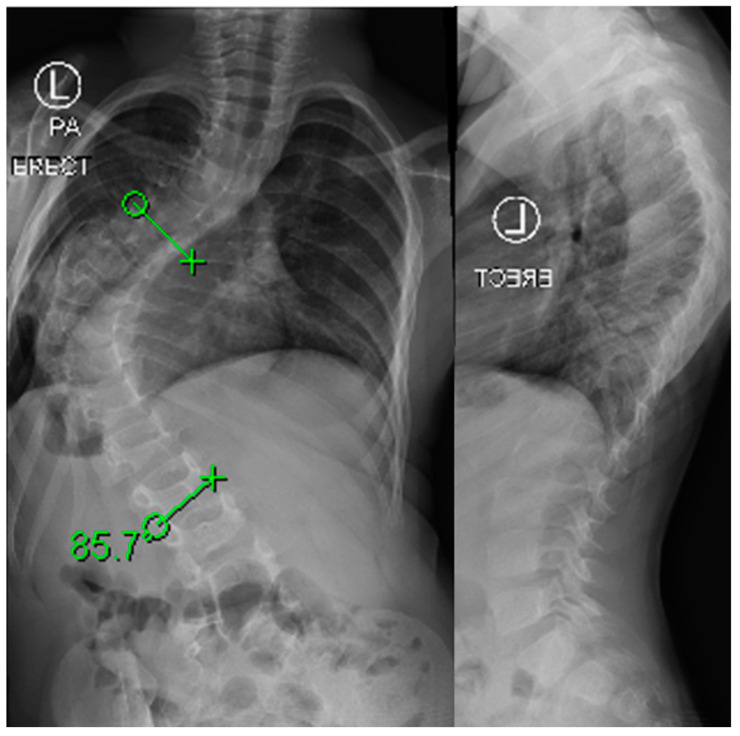
Preoperative images of a 3-year-old male with progression despite prolonged casting.

**Figure 5:**
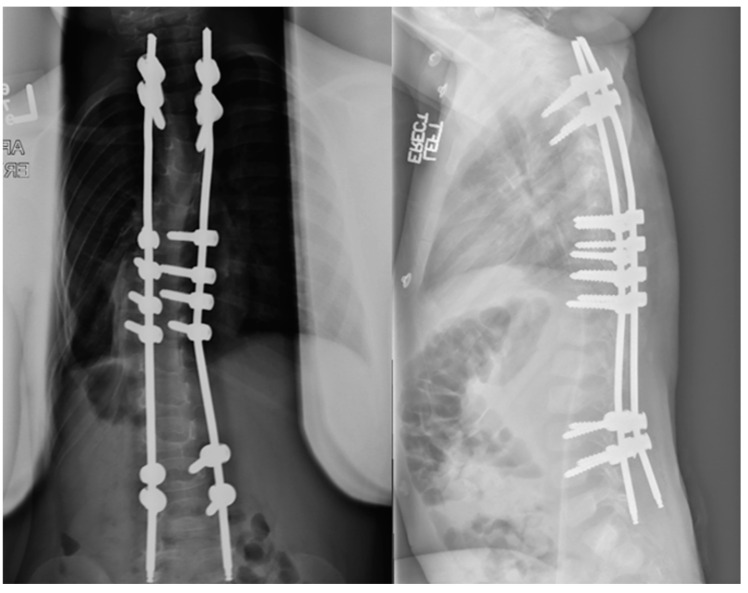
The same patient at six weeks after instrumentation utilizing Shilla technique.

One mechanical disadvantage of distraction-based constructs is lack of apical control. Luque et al. described a “trolley” technique allowing spinal growth by rods stabilized with sublaminar wires to glide during growth [54]. The subperiosteal dissection required for this technique unfortunately led to autofusion in many cases. In 2014, McCarthy et al. described a technique which they termed the “Shilla technique” employing a short apical fusion with rods left long through sliding screws at the proximal and distal aspects [55]. This allows the remainder of the spine to grow proximally and distally, obviating the need for repeated lengthening surgeries. A clinical example is shown pre-operatively in Figure 4 and post-operatively in Figure 5. While this technique has shown promise, it is not suitable for all curve patterns. Additionally, rod breakage is common adjacent to the fused segment but typically takes a few years to occur.

In 2003, Betz et al. described the first series of vertebral body stapling for growth modulation. Initial, short-term studies showed promising results with curve improvement in 79–100% of patients with lower magnitude (<35 degrees) and lumbar curves having more favorable outcomes [56,57,58]. More recent studies do raise longevity concerns and no superiority over bracing in most patients [59]. Longer-term results were published by Trupia in 2019 which demonstrated that despite early curve correction, it did not prevent long-term curve progression. Specifically, it did not affect the percentage of patients who progressed to fusion [60].

**Figure 6:**
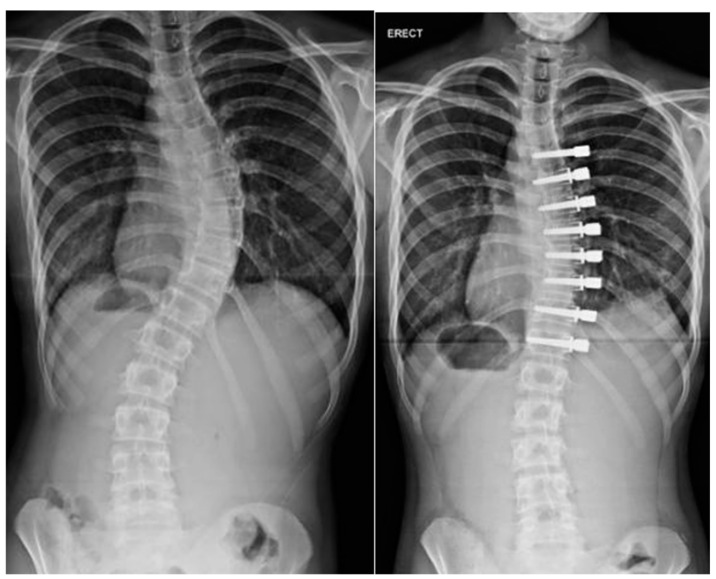
Pre- and postoperative imaging of a female patient with early onset scoliosis treated with anterior vertebral body tethering.

Initially developed for idiopathic scoliosis curves from 45–60 degrees, vertebral body tethering has recently been explored for older early onset patients [61,62,63]. In this population of 8–11 with Sanders scores less than four, dubbed “Tweeners”, VBT was shown to be as effective as MCGRs and PSF for correction of deformity. A clinical example is shown in Figure 6. In a study supplying direct comparison amongst the three, major scoliosis curve correction was seen in all three groups including by 41.1% with VBT, 52.2% with PSF, and by 27.4% with MCGRs. This same study demonstrated high rates of unplanned operations in MCGRs compared to PSF and VBT with 21.6% of MCGR, 16.2% of VBT, and 7.1% of PSF patients undergoing at least one unplanned revision. Technical considerations exist for VBT including instrumentation sizes that may not be appropriate for the smallest patients. Applying VBT to younger patients may not fully prevent the need for definitive fusion, however, it could prevent the progression of lumbar curves and make selective thoracic fusions more successful and limit lumbar instrumentation, although this is largely theoretical and should not diminish the morbidity of a secondary surgery. VBT does confer technique-specific complications including those associated with thorascopic and anterior surgery, overcorrection, and the risk of cord breakage which is seen in up to 50% of thoracic tethers and is as high as 70–80% in the lumbar spine [64]. While there are few studies examining the application of tethering in the early onset population, available data suggest it is an option in older early onset patients.

Additionally, another modern fusionless technique is the posterior dynamic distraction device (ApiFix^®^, Orthopediatrics Corp., Warsaw, IN, USA). In this novel short segment pedicle screw-based instrumentation, the device is inserted around the apex of the main curve. The system has a ratchet mechanism that enables gradual postoperative device elongation and curve correction. Again, this device is primarily indicated in adolescent idiopathic scoliosis but, as a fusionless technique, it will likely be expanded into the early onset population. Clinical trials are ongoing and future investigation will be necessary prior to widespread implementation.

In children with congenital scoliosis, one may consider a convex hemiepiphysiodesis. This procedure requires an anterior and posterior approach in an attempt to cease growth on the convex side of the curve. While the technique seems promising in very young children, the growth modulation affect is not as robust in children over age 8 [65].

## 11. Definitive Fusion

Historically, early definitive posterior arthrodesis was felt to be the most appropriate treatment for early onset scoliosis [66]. Long-term follow-up studies demonstrated that revision rates approach 40% of early fusion patients [67,68]. Even more concerning is thoracic insufficiency syndrome and the associated restrictive pulmonary pattern seen as a result of fusion with FVC < 50% of normal. In the landmark paper related to this, Karol et al. revealed the importance of a T1–T12 thoracic height of greater than 18 cm [68]. Unfortunately, longer-term follow-up of these patients that achieved the 18 cm threshold, even with ~50° residual curve, does not reliably stabilize the long-term pulmonary prognosis for the patient with EOS [69]. Preserving spinal and thoracic growth while controlling deformity remains a central goal of treatment.

Short fusions can be effective in the treatment of congenital scoliosis, especially when combined with hemivertebra resection. Indications include progressive curves, or those known from natural history to be at high risk for progression such as a hemivertebra at the lumbosacral junction, or a hemivertebra with a contralateral bar [65].

## 12. Conclusions

Early onset scoliosis is a challenging diagnosis to treat with significant equipoise remaining in many domains. Treatment approaches vary based on the etiology, severity of the curve, and age. In all cases, protecting growth of the chest wall, controlling deformity, optimizing comorbidities and health-related quality of life, and delaying surgical interventions are central to the treatment strategy. Due to the heterogeneity of presentations, there is no single or algorithmic approach that can be applied to early onset scoliosis.

- The C-EOS is a useful tool in categorizing and understanding risk in patients with early onset scoliosis.
- Recent expert consensus was reached for conservative management in all patients aged 3 or younger with preference to casting and has reinforced avoidance of surgery whenever possible.
- Medical optimization of children with EOS is of utmost importance prior to any surgical management with particular attention to nutrition and pulmonary strategies.
- Consensus for surgical intervention was reached for EOS patients older in age (6- or 9-year-olds) with increased (30°) curve progression, greater curve degree, and those with rigid curves. Distraction-based systems remain highly favored in young patients with less enthusiasm in older children [70].

Treatment of early onset scoliosis can be incredibly rewarding. This challenging condition requires open communication and treatment goal awareness amongst a multidisciplinary team.

## Figures and Tables

**Table 1:** Common Causes of Early Onset Scoliosis.

Idiopathic	Syndromic	Neuromuscular	Congenital
Infantile Juvenile	Jarcho–Levine Marfan’s VACTER Goldenhar Spinal Muscular Atrophy Prader–Willi Neurofibromatosis Skeletal Dysplasia	Cerebral Palsy Spina Bifida Muscular Dystrophy Spinal Cord Injury Chiari Malformation Syringomyelia	Failure of Formation Failure of Segmentation Mixed Failure of Segmentation/Formation

## References

[1] Williams B.A., Matsumoto H., McCalla D.J., Akbarnia B.A., Blakemore L.C., Betz R.R., Flynn J.M., Johnston C.E., McCarthy R.E., Roye D.P. (2014). Development and initial validation of the Classification of Early-Onset Scoliosis (C-EOS). J. Bone Jt. Surg. Am..

[2] Cyr M., Hilaire T.S., Pan Z., Thompson G.H., Vitale M.G., Garg S. (2017). Classification of early onset scoliosis has excellent interobserver and intraobserver reliability. J. Pediatr. Orthop..

[3] Mehta M.H. (2005). Growth as a corrective force in the early treatment of progressive infantile scoliosis. J. Bone Jt. Surg. Br..

[4] Fedorak G.T., D’Astous J.L., Nielson A.N., MacWilliams B.A., Heflin J.A. (2019). Minimum 5-Year Follow-up of Mehta Casting to Treat Idiopathic Early-Onset Scoliosis. J. Bone Jt. Surg. Am..

[5] Fedorak G.T., MacWilliams B.A., Stasikelis P., Szczodry M., Lerman J., Pahys J.M., D’Astous J. (2022). Age-Stratified Outcomes of Mehta Casting in Idiopathic Early-Onset Scoliosis: A Multicenter Review. J. Bone Jt. Surg. Am..

[6] Welborn M.C., D’Astous J., Bratton S., Heflin J. (2018). Infantile Idiopathic Scoliosis: Factors Affecting EDF Casting Success. Spine Deform..

[7] Demirkiran H.G., Bekmez S., Celilov R., Ayvaz M., Dede O., Yazici M. (2015). Serial derotational casting in congenital scoliosis as a time-buying strategy. J. Pediatr. Orthop..

[8] Cao J., Zhang X.-J., Sun N., Sun L., Guo D., Qi X.-Y., Bai Y.-S., Sun B.-S. (2017). The therapeutic characteristics of serial casting on congenital scoliosis: A comparison with non-congenital cases from a single-center experience. J. Orthop. Surg. Res..

[9] LaValva S.A.A., MacAlpine E., Gupta P., Hammerberg K., Thompson G.H., Sturm P., Garg S., Anari J., Sponseller P., Flynn J. (2020). Serial Casting in Neuromuscular and Syndromic Early-onset Scoliosis (EOS) Can Delay Surgery Over 2 Years. J. Pediatr. Orthop..

[10] Fletcher N.D., McClung A., Rathjen K.E., Denning J.R., Browne R., Johnston C.E. (2012). Serial casting as a delay tactic in the treatment of moderate-to-severe early-onset scoliosis. J. Pediatr. Orthop..

[11] Gomez J.A., Grzywna A., Miller P.E., Karlin L.I., Garg S., Sanders J.O., Sturm P.F., Sponseller P.D., D’Astous J.L., Glotzbecker M.P. (2017). Initial Cast Correction as a Predictor of Treatment Outcome Success for Infantile Idiopathic Scoliosis. J. Pediatr. Orthop..

[12] Fedorak G.T., Stasikelis P.J., Carpenter A.M., Nielson A.N., D’Astous J.L. (2019). Optimization of Casting in Early-onset Scoliosis. J. Pediatr. Orthop..

[13] Montgomery B.K., Tileston K., Kaur J., Kym D., Segovia N.A., Imrie M., Policy J., Rinsky L., Vorhies J. (2022). Innovative technique for early-onset scoliosis casting using Jackson table. Spine Deform..

[14] LaValva S.M., MacAlpine E.M., Kawakami N., Gandhi J.S., Morishita K., Sturm P.F., Garg S., Glotzbecker M.P., Anari J.B., Pediatric Spine Study Group (2020). Awake serial body casting for the management of infantile idiopathic scoliosis: Is general anesthesia necessary?. Spine Deform..

[15] Thometz J., Liu X., Rizza R., English I., Tarima S. (2018). Effect of an elongation bending derotation brace on the infantile or juvenile scoliosis. Scoliosis Spinal Disord..

[16] Thometz J., Liu X.C. (2019). Serial CAD/CAM Bracing: An Alternative to Serial Casting for Early Onset Scoliosis. J. Pediatr. Orthop..

[17] Negrini S., Donzelli S., Jurenaite G., Negrini F., Zaina F. (2021). Efficacy of bracing in early infantile scoliosis: A 5-year prospective cohort shows that idiopathic respond better than secondary-2021 SOSORT award winner. Eur. Spine J..

[18] Weinstein S.L. (2022). Casting vs. Bracing for Idiopathic Early-Onset Scoliosis (CVBT).

[19] Minsk M.K., Venuti K.D., Daumit G.L., Sponseller P.D. (2017). Effectiveness of the Rigo Chêneau versus Boston-style orthoses for adolescent idiopathic scoliosis: A retrospective study. Scoliosis Spinal Disord..

[20] Williams B.A., McClung A., Blakemore L.C., Shah S.A., Pawelek J.B., Sponseller P.D., Parent S., Emans J.B., Sturm P.F., The Pediatric Spine Study Group (2020). MRI utilization and rates of abnormal pretreatment MRI findings in early-onset scoliosis: Review of a global cohort. Spine Deform..

[21] Tong Y., Udupa J.K., McDonough J.M., Wileyto E.P., Capraro A., Wu C., Ho S., Galagedera N., Talwar D., Mayer O.H. (2019). Quantitative Dynamic Thoracic MRI: Application to Thoracic Insufficiency Syndrome in Pediatric Patients. Radiology.

[22] Udupa J.K., Tong Y., Capraro A., McDonough J.M., Mayer O.H., Ho S., Wileyto P., Torigian D.A., Campbell R.M. (2020). Understanding Respiratory Restrictions as a Function of the Scoliotic Spinal Curve in Thoracic Insufficiency Syndrome: A 4D Dynamic MR Imaging Study. J. Pediatr. Orthop..

[23] Mohanty S.P., Kanhangad M.P., Kurup J.K.N., Saiffudeen S. (2020). Vertebral, intraspinal and other organ anomalies in congenital scoliosis. Eur. Spine J..

[24] Wu N., Liu L., Zhang Y., Wang L., Wang S., Zhao S., Li G., Yang Y., Lin G., Shen J. (2023). Retrospective Analysis of Associated Anomalies in 636 Patients with Operatively Treated Congenital Scoliosis. J. Bone Jt. Surg Am..

[25] Mayer O.H., Redding G. (2009). Early changes in pulmonary function after vertical expandable prosthetic titanium rib insertion in children with thoracic insufficiency syndrome. J. Pediatr. Orthop..

[26] Redding G.J., Mayer O.H. (2011). Structure-respiration function relationships before and after surgical treatment of early-onset scoliosis. Clin. Orthop. Relat. Res..

[27] Nakashima H., Kawakami N.M., Matsumoto H., Redding G.J. (2020). Preoperative 6-Minute Walk Performance in Children With Congenital Scoliosis. J. Pediatr. Orthop..

[28] Striegl A., Chen M.L., Kifle Y., Song K., Redding G. (2010). Sleep-disordered breathing in children with thoracic insufficiency syndrome. Pediatr. Pulmonol..

[29] Skaggs D.L., Akbarnia B.A., Flynn J.M., Myung K.S., Sponseller P.D., Vitale M.G., Chest Wall and Spine Deformity Study Group, Growing Spine Study Group, Pediatric Orthopaedic Society of North America, Scoliosis Research Society Growing Spine Study Committee (2014). A classification of growth friendly spine implants. J. Pediatr. Orthop..

[30] Welborn M.C., Krajbich J.I., D’Amato C. (2019). Use of Magnetic Spinal Growth Rods (MCGR) With and Without Preoperative Halo-gravity Traction (HGT) for the Treatment of Severe Early-onset Scoliosis (EOS). J. Pediatr. Orthop..

[31] Iyer S., Duah H.O., Wulff I., Osei Tutu H., Mahmud R., Yankey K.P., Akoto H., Boachie-Adjei O., FOCOS Spine Research Group (2019). The Use of Halo Gravity Traction in the Treatment of Severe Early Onset Spinal Deformity. Spine.

[32] Yang J.S., McElroy M.J., Akbarnia B.A., Salari P., Oliveira D., Thompson G.H., Emans J.B., Yazici M., Skaggs D.L., Shah S.A. (2010). Growing rods for spinal deformity: Characterizing consensus and variation in current use. J. Pediatr. Orthop..

[33] Bess S., Akbarnia B.A., Thompson G.H., Sponseller P.D., Shah S.A., El Sebaie H., Boachie-Adjei O., Karlin L.I., Canale S., Poe-Kochert C. (2010). Complications of growing-rod treatment for early-onset scoliosis: Analysis of one hundred and forty patients. J. Bone Joint Surg. Am..

[34] Sewell M.D., Malagelada F., Wallace C., Gibson A., Noordeen H., Tucker S., Molloy S., Lehovsky J. (2016). A Preliminary Study to Assess Whether Spinal Fusion for Scoliosis Improves Carer-assessed Quality of Life for Children With GMFCS Level IV or V Cerebral Palsy. J. Pediatr. Orthop..

[35] Akbarnia B.A., Breakwell L.M., Marks D.S., McCarthy R.E., Thompson A.G., Canale S.K., Kostial P.N., Tambe A., Asher M.A., Growing Spine Study Group (2008). Dual growing rod technique followed for three to eleven years until final fusion: The effect of frequency of lengthening. Spine.

[36] Thompson G.H., Akbarnia B.A., Kostial P., Poe-Kochert C., Armstrong D.G., Roh J., Lowe R., Asher M.A., Marks D.S. (2005). Comparison of single and dual growing rod techniques followed through definitive surgery: A preliminary study. Spine.

[37] Varley E.S., Pawelek J.B., Mundis G.M., Oetgen M.E., Sturm P.F., Akbarnia B.A., Yaszay B., Pediatric Spine Study Group (2021). The role of traditional growing rods in the era of magnetically controlled growing rods for the treatment of early-onset scoliosis. Spine Deform..

[38] Takaso M., Moriya H., Kitahara H., Minami S., Takahashi K., Isobe K., Yamagata M., Otsuka Y., Nakata Y., Inoue M. (1998). New remote-controlled growing-rod spinal instrumentation possibly applicable for scoliosis in young children. J. Orthop. Sci..

[39] Akbarnia B.A., Pawelek J.B., Cheung K.M.C., Demirkiran G., Elsebaie H., Emans J.B., Johnston C.E., Mundis G.M., Noordeen H., Skaggs D.L. (2014). Traditional growing rods versus magnetically controlled growing rods for the surgical treatment of early-onset scoliosis: A case-matched 2-year study. Spine Deform..

[40] Lebel D.E., Rocos B., Helenius I., Sigal A., Struder D., Yazici M., Bekmez S., Hasler C.C., Pesenti S., Jouve J.L. (2021). Magnetically controlled growing rods graduation: Deformity control with high complication rate. Spine.

[41] Thakar C., Kieser D.C., Mardare M., Haleem S., Fairbank J., Nnadi C. (2018). Systematic review of the complications associated with magnetically controlled growing rods for the treatment of early onset scoliosis. Eur. Spine J..

[42] Zhang T., Sze K.Y., Peng Z.W., Cheung K.M., Lui Y.F., Wong Y.W., Kwan K.Y., Cheung J.P. (2020). Systematic investigation of metallosis associated with magnetically controlled growing rod implantation for early-onset scoliosis. Bone Jt J..

[43] McIntosh A.L., Booth A., Oetgen M.E. (2024). Unplanned return to the operating room (UPROR) occurs in 40% of MCGR patients at an average of 2 years after initial implantation. Spine Deform.

[44] Matsumoto H., Skaggs D.L., Akbarnia B.A., Pawelek J.B., Hilaire T.S., Levine S., Sturm P., Perez-Grueso F.J.S., Luhmann S.J., Sponseller P.D. (2021). Comparing health-related quality of life and burden of care between early-onset scoliosis patients treated with magnetically controlled growing rods and traditional growing rods: A multicenter study. Spine Deform..

[45] Sankar W.N., Skaggs D.L., Yazici M., Johnston C.E., Shah S.A., Javidan P., Kadakia R.V., Day T.F., Akbarnia B.A. (2010). Lengthening of dual growing rods and the Law of Diminishing Returns. Spine.

[46] Heyer J.H., Anari J.B., Baldwin K.D., Mitchell S.L., Luhmann S.J., Sturm P.F., Flynn J.M., Cahill P.J., on behalf of the Pediatric Spine Study Group (2022). Lengthening Behavior of Magnetically Controlled Growing Rods in Early-Onset Scoliosis: A Multicenter Study. J. Bone Jt. Surg..

[47] Doany M.E., Olgun Z.D., Kinikli G.I., Bekmez S., Kocyigit A., Demirkiran G., Karaagaoglu A.E., Yazici M. (2018). Health-Related Quality of Life in Early-Onset Scoliosis Patients Treated Surgically: EOSQ Scores in Traditional Growing Rod Versus Magnetically Controlled Growing Rods. Spine.

[48] Klyce W., Mitchell S.L., Pawelek J., Skaggs D.L., Sanders J.O., Shah S.A., McCarthy R.E., Luhmann S.J., Sturm P.F., Flynn J.M. (2020). Characterizing use of growth-friendly implants for early-onset scoliosis: A 10-year update. J. Pediatr. Orthop..

[49] Campbell R.M., Smith M.D., Mayes T.C., Mangos J.A., Willey-Courand D.B., Kose N., Pinero R.F., Alder M.E., Duong H.L., Surber J.L. (2004). The effect of opening wedge thoracostomy on thoracic insufficiency syndrome associated with fused ribs and congenital scoliosis. J. Bone Jt. Surg. Am..

[50] El-Hawary R., Morash K., Kadhim M., Vitale M., Smith J., Samdani A., Flynn J. (2020). VEPTR Treatment of Early Onset Scoliosis in Children Without Rib Abnormalities: Long-term Results of a Prospective, Multicenter Study. J. Pediatr. Orthop..

[51] Waldhausen J.H., Redding G., White K., Song K. (2016). Complications in using the vertical expandable prosthetic titanium rib (VEPTR) in children. J. Pediatr. Surg..

[52] Wijdicks S.P.J., Tromp I.N., Yazici M., Kempen D.H.R., Castelein R.M., Kruyt M.C. (2019). A comparison of growth among growth-friendly systems for scoliosis: A systematic review. Spine J..

[53] Tabeling C.S., Lemans J.V.C., Top A., Scholten E.P., Stempels H.W., Schlösser T.P.C., Ito K., Castelein R.M., Kruyt M.C. (2022). The Spring Distraction System for Growth-Friendly Surgical Treatment of Early Onset Scoliosis: A Preliminary Report on Clinical Results and Safety after Design Iterations in a Prospective Clinical Trial. J. Clin. Med..

[54] Luqué E.R., Cardoso A. (1977). Treatment of scoliosis without arthrodesis or external support, preliminary report. Orthop. Trans..

[55] McCarthy R.E., Luhmann S., Lenke L., McCullough F.L.R. (2014). The Shilla Growth Guidance Technique for Early-Onset Spinal Deformities at 2-Year Follow-Up: A Preliminary Report. J. Pediatr. Orthop..

[56] Betz R.R., Kim J., D’Andrea L.P., Mulcahey M.J., Balsara R.K., Clements D.H. (2003). An innovative technique of vertebral body stapling for the treatment of patients with adolescent idiopathic scoliosis: A feasibility, safety, and utility study. Spine.

[57] Betz R.R., Ranade A., Samdani A.F., Chafetz R., D’andrea L.P., Gaughan J.P., Asghar J., Grewal H., Mulcahey M.J. (2010). Vertebral body stapling: A fusionless treatment option for a growing child with moderate idiopathic scoliosis. Spine.

[58] Trobisch P.D., Samdani A., Cahill P., Betz R.R. (2011). Vertebral body stapling as an alternative in the treatment of idiopathic scoliosis. Oper. Orthop. Traumatol..

[59] Cuddihy L., Danielsson A.J., Cahill P.J., Samdani A.F., Grewal H., Richmond J.M., Mulcahey M.J., Gaughan J.P., Antonacci M.D., Betz R.R. (2015). Vertebral body stapling versus bracing for patients with high-risk moderate idiopathic scoliosis. Biomed Res. Int..

[60] Trupia E., Hsu A.C., Mueller J.D., Matsumoto H., Bodenstein L., Vitale M. (2019). Treatment of idiopathic scoliosis with vertebral body stapling. Spine Deform..

[61] Newton P.O. (2020). Spinal growth tethering: Indications and limits. Ann. Transl. Med..

[62] Baroncini A., Courvoisier A. (2023). The different applications of Vertebral Body Tethering—Narrative review and clinical experience. J. Orthop..

[63] Mackey C., Hanstein R., Lo Y., Vaughan M., St Hilaire T., Luhmann S.J., Vitale M.G., Glotzbecker M.P., Samdani A., Parent S. (2022). Magnetically Controlled Growing Rods (MCGR) Versus Single Posterior Spinal Fusion (PSF) Versus Vertebral Body Tether (VBT) in Older Early Onset Scoliosis (EOS) Patients: How Do Early Outcomes Compare?. Spine.

[64] Cahill P.J., Miyanji F., Lullo B.R., Samdani A.F., Lonner B.S., Pahys J.M., Hwang S.W., Haber L.L., Alanay A., Shah S.A. (2024). Incidence of Tether Breakage in Anterior Vertebral Body Tethering. J. Pediatr. Orthop..

[65] Hedequist D.J. (2007). Surgical treatment of congenital scoliosis. Orthop. Clin. North Am..

[66] Winter R.B., Moe J.H. (1982). The results of spinal arthrodesis for congenital spinal deformity in patients younger than five years old. J. Bone Jt. Surg. Am..

[67] Goldberg C.J., Moore D.P., Fogarty E.E., Dowling F.E. (2002). Long-term results from in situ fusion for congenital vertebral deformity. Spine.

[68] Karol L.A., Johnston C., Mladenov K., Schochet P., Walters P., Browne R.H. (2008). Pulmonary function following early thoracic fusion in non-neuromuscular scoliosis. J. Bone Jt. Surg. Am..

[69] Johnston C.E., Karol L.A., Thornberg D., Jo C., Eamara P. (2021). The 18-cm Thoracic-Height Threshold and Pulmonary Function in Non-Neuromuscular Early-Onset Scoliosis: A Reassessment. JB JS Open Access..

[70] Matsumoto H., Fano A.N., Quan T., Akbarnia B.A., Blakemore L.C., Flynn J.M., Skaggs D.L., Smith J.T., Snyder B.D., Sponseller P.D. (2023). Re-evaluating consensus and uncertainty among treatment options for early onset scoliosis: A 10-year update. Spine Deform..

